# Post-exercise left ventricular dysfunction measured after a long-duration cycling event

**DOI:** 10.1186/1756-0500-6-211

**Published:** 2013-05-26

**Authors:** Enrique Serrano Ostariz, Marta López Ramón, Daniel Cremades Arroyos, Silvia Izquierdo Álvarez, Pilar Catalán Edo, Cristina Baquer Sahún, Alejandro Legaz Arrese

**Affiliations:** 1 Physical Education and Sports Section, Universidad de Zaragoza, Zaragoza, Spain; 2 Cardiology Service, Hospital Universitario Miguel Servet, Zaragoza, Spain; 3 Servicio de Bioquímica Clínica Planta 3a, Edificio de Consultas Externas, Hospital Universitario Miguel Servet, Calle Padre Arrupe, s/n, Zaragoza 50009, Spain; 4 Hospital Clínico Universitario Lozano Blesa, Zaragoza, Spain

**Keywords:** Cycling event, Cardiac biomarkers, Prolonged exercise, Strenuous exercise

## Abstract

**Background:**

In this research, an extension to our previous work published in the *Clinical Journal of Sports Medicine* in 2009, we studied subjects that differed in terms of age and training status and assessed the impact of prolonged exercise on systolic and left ventricular diastolic function and cardiac biomarkers levels, recognized as identifiers of cardiac damage and dysfunction. We also assessed the possible influence of event duration, exercise intensity and weight loss (dehydration) on left ventricular diastolic function.

**Findings:**

Ninety-one male cyclists were assessed by echocardiography and serum biomarkers before and after the 2005 Quebrantahuesos cycling event (206 km long and with an accumulated slope of 3800 m). Cardiac function was assessed by echocardiography and cardiac biomarkers were assessed in blood serum. Echocardiograms measured left ventricular internal dimension during diastole and systole, left ventricular posterior wall thickness during diastole, interventricular septum thickness during diastole, left ventricular ejection fraction and diastolic filling. The heart rate of 50 cyclists was also monitored during the race to evaluate exercise intensity. Echocardiograph results indicated that left ventricular diastolic and systolic function decreased after the race, with systolic function reduced to a significant degree. Left ventricular ejection fraction was below 55% in 29 cyclists. The decrease in left ventricular systolic and diastolic function did not correlate with age, training status, race duration, weight loss or exercise intensity.

**Conclusions:**

Left ventricular systolic and diastolic function was reduced and cardiac biomarkers were increased after the cycling event, but the mechanisms behind such outcomes remain unclear.

## Findings

### Background

This study is a further analysis of data collected for a previous work [1]. One potential effect of prolonged strenuous exercise is exercise-induced cardiac fatigue, described as an alteration to ventricular systolic or diastolic function following such exercise [2,3]. Findings reported for numerous ventricular dysfunction studies have often been inconsistent [3-8], with disparities possibly due to small sample sizes, differences in exercise intensity and duration and great variability in the training status of the study subjects. As for cardiac-specific biomarkers of damage and dysfunction, a growing number of studies have demonstrated that prolonged and strenuous exercise raises cardiac troponin T and I (cTnT and cTnI, respectively) and N-terminal pro-brain natriuretic peptide (NT-proBNP) levels [9-14]. The link between systolic and diastolic ventricular dysfunction and the increase in biomarkers is not clear, however [15-18]. Most studies of high-intensity prolonged exercise resulting in increased cardiac biomarkers have been performed in relatively old subjects, but increases are not so evident for elite or professional sportspeople [19,20].

Specific exercise parameters (training status and exercise intensity and duration) that may affect observed changes in biomarkers and ventricular systolic and diastolic function are poorly described and the mechanisms involved are not well understood. To the best of our knowledge, the influence of exercise intensity on left ventricular function has not yet been determined, as we were unable to locate any studies of ventricular dysfunction in cyclists of different ages and training levels for a long-duration cycling event (6 hours or more). Therefore, for a long-duration cycling test, we studied, following a similar study [19], the influence of age, level of training, exercise intensity, event duration and weight loss (dehydration) on ventricular function and their association with cardiac biomarkers.

## Methods

### Participants and study design

The study protocol was approved by the Research Ethics Committee of the Government of Aragón (Spain). Amateur male cyclists scheduled to participate in the 2005 Quebrantahuesos Cycle Race (UCI Golden Bike series) were invited to participate in the study via the official race organizer’s website [1]. The first 95 volunteers were recruited and their written consent was obtained (note that sample size was limited by the finishing-line resources available). Since 4 cyclists dropped out during the race, the study finally included 91 cyclists, profiled as follows in terms of mean ± standard deviation (SD): age, 40 ± 9 years; height, 1.76 ± 0.07 m; weight, 74.1 ± 8.4 kg; and training experience, 12 ± 7 years [1]. The race workload (total distance 206 km for an accumulated slope of 3800 m) was comparable to that of the tougher mountain stages of the Tour de France. Ambient temperature during the test ranged between 16°C and 32.5°C.

Subjects completed a general questionnaire, to ascertain personal and cycling history, and a log referring to cycle training in the previous 6 months (4358 ± 1731 km). Cyclists were assessed the day before, and approximately 20 minutes after, the cycling event [1]. Cyclists consumed fluid during the event, but, in accordance with guidelines by White et al. [21], consumption was not allowed until after post-race data was collected so as not to influence left ventricular loading.

Pre- and post-race assessment of the 91 cyclists included the following: echocardiography, body mass, heart rate (HR), and blood pressure measurements and a 12-lead electrocardiogram. HR for 50 of the cyclists was also measured during the race using a Polar HR monitor (Polar Team System, Polar Electro Oy, Finland) and the data was downloaded using Polar Precision Performance software (version 3.0) [1]. Maximum HR was calculated using the standard equation HR (max) = (220-age). Exercise intensity was defined by the HR ratio as HR (average)/HR (max) [22]. Finally, training impulse (TRIMP) values, which are used as an integrative marker of exercise load during competition, were calculated from event duration and the average HR using the formula described by Banister [23].

### Echocardiograph procedures

Participants underwent a resting echocardiographic examination in the left lateral decubitus position. One experienced sonographer performed all the measurements using a commercially available system (ATL ultrasound HDI 5000) and a 2.5 MHz phased array transducer. Two-dimensionally guided M-mode echocardiograms were obtained from the left parasternal long-axis view. These recordings, made according to American Society of Echocardiography recommendations [24], were used to obtain the following measurements: left ventricular internal dimension during diastole and systole (LVIDd and LVIDs, respectively), left ventricular posterior wall thickness during diastole (LVPWd) and interventricular septum thickness during diastole (IVSDd). Left ventricular ejection fraction (LVEF, %) was determined by calculating left ventricular volumes according to Teichholz et al. [25]. Doppler echocardiography was used to assess diastolic function. An apical four-chamber view was obtained for a maximized diameter of the mitral annulus. With the sample volume cursor aligned parallel to flow at the mitral annulus level, inflow velocities were interrogated by pulsed-wave Doppler. Minor adjustments were made to the transducer to ensure optimal spectral display, that is, the highest velocity for the least spectral dispersion. The velocity curves were digitized through the darkest grey scale and averages were calculated for the measurements obtained. Peak early and late diastolic filling (E and A, respectively) were measured and the corresponding ratios calculated. HR was determined by limb-lead electrocardiography combined with echocardiography. Blood pressure was simultaneously measured using standard auscultation procedures.

### Blood sampling procedures

Procedures were as described in the previous work published [1]. The upper reference limit (URL) for cTnI (defined as the 99th percentile of healthy participants) was less than 0.04 μg L-1 [26]. NT-proBNP levels were measured using an electrochemiluminescence sandwich immunoassay (Elecsys ProBNP, Roche Diagnostics) with the Roche 2010 system. The URL was set at 125 ng L-1 [27]. Haematocrit concentrations were measured using an automated analyser (Sysmex K-1000; Sysmex GmbH, Langenfeld, Germany).

### Statistical analyses

Statistical analyses were performed using SPSS version 14.0. Data were expressed as means and standard deviations. Paired student’s t-tests were used to determine differences between pre- and post-race mean values for the various echocardiographic parameters. Analyses for non-Gaussian distributed variables were performed using the Wilcoxon test for paired samples.

Pearson and Spearman correlation coefficients were calculated as standard. Multivariate linear regression was used to assess the relationships between left ventricular diastolic function — measured as the dependent variable — and age, weight loss, training status, event duration and exercise intensity as independent predictors. The level of significance was set to *P* < 0.05.

## Results

All 91 cyclists lost weight (74.2 ± 8.6 kg pre-race weight versus 71.8 ± 8.6 kg post-race weight; *P* < 0.001) [1]. Post-race systolic blood pressure was lower (from 124 ± 12 mm Hg to 110 ± 11 mm Hg; *P* < 0.001) [1]. No cyclist required medical attention.

### Echocardiographic data

Baseline echocardiographic examinations revealed no remarkable pathological findings. No cyclist had LVPWd or IVSDd values of more than 12 mm. Mean LVIDd was 51.2 ± 4.0 mm, mean LVEF (%) was 67.7 ± 8.2 and the mean E/A ratio was 1.6 ± 0.4.

The cycling event exercise resulted in decreased LVIDd and increased LVIDs in the participants but produced no change in the LVPWd or IVSDd (Table 1). The LVEF (%) was reduced from 67.7 ± 8.2 to 59.7 ± 9.4 (*P* < 0.001) and, in 29 cyclists, post-race LVEF was below 55%.

**Table 1:** **Pre and post**-**race data** (**mean** ± **standard deviation**)

	**Pre**-**race**	**Post**-**race**	***P***
LVIDd (mm)	51.2 ± 4.0	47.7 ± 4.6	0.000
LVIDs (mm)	29.3 ± 4.1	30.4 ± 4.3	0.018
LVPWd (mm)	9.6 ± 1.1	9.6 ± 0.9	0.80
IVSDd (mm)	9.6 ± 1.1	9.5 ± 1.0	0.58
LVEF (%)	67.7 ± 8.2	59.7 ± 9.4	0.000
E/A	1.6 ± 0.4	1.1 ± 0.3	0.000
cTnI (μg L-1)	0.006 ± 0.015	0.056 ± 0.059	0.000
NT-proBNP (ng L-1)	27 ± 16	189 ± 111	0.000
HR (per min)	58 ± 8	86 ± 11	0.000
BPS (mmHg)	125 ± 12	110 ± 12	0.000
BPD (mmHg)	65 ± 10	62 ± 111	0.049
Ht (%)	43.3 ± 2.1	44.6 ± 2.9	0.000
Weight (kg)	74.3 ± 8.4	72.0 ± 8.5	0.000

Abbreviations: *BPD* diastolic blood pressure, *BPS* systolic blood pressure, *cTnI* cardiac troponin I, *E*/*A* ratio of early to late peak diastolic transmitral flow velocities, *H R* heart rate, *Ht* haematocrit, *IVSDd* interventricular septum thickness during diastole, *LVEF* left ventricular ejection fraction, *LVIDd* left ventricular internal dimension during diastole, *LVIDs* left ventricular internal dimension during systole, *LVPWd* left ventricular posterior wall thickness during diastole, *NT*-*proBNP* N-terminal pro-brain natriuretic peptide.

Early transmitral diastolic filling velocities were reduced and late transmitral diastolic filling velocities were increased, resulting in a reduced E/A ratio (Table 1). The post-race increase in HR was not significantly associated with alterations in the LVEF and the E/A ratio. The LVIDd was not related to any of the observed functional changes. Overall, there were no significant associations between alterations in cardiac function (systolic or diastolic) and age, training status, event duration, weight loss, exercise intensity (the HR (average)/HR (max) ratio or TRIMP values) and NT-proBNP. When we considered multivariate regression, the model was not significant (systolic: R^2^ = 0.147, *P* = 0.267; diastolic: R^2^ = 0.091, *P* = 0.599).

### Biochemical markers

Post-race cTnI and NT-proBNP levels were significantly raised, with 43% of participants exhibiting cTnI levels above 0.04 μg L-1 and with 65% of the participants exhibiting NT-proBNP levels above 125 ng L-1 [1]. The increases, however, did not correlate with post-race cardiac function.

## Discussion

The results of this study confirm those of previous studies (running and ironman) in demonstrating that prolonged strenuous exercise reduces systolic and diastolic function [6,21,28-30] and increases the cardiac-specific markers cTnI and NT-proBNP [5,10,13,14,30,31].

The decrease in post-race LVEF supports the suggestion that systolic changes only develop during long-duration events [5,12,15,21,32,33].

No previous studies have been conducted for amateur cyclists of different ages and training levels or for long-duration cycling events (more than 6 hours). The long duration of the event may explain the deterioration in ventricular function in most cyclists in our study, as this finding coincides with the deterioration reported in studies for other longer duration tests.

The decrease in LVEF in the presence of decreased systolic blood pressure reflects depressed cardiac contractility. The lack of correlation between weight changes, LVIDd and diastolic function measurements suggests that the depression in diastolic function represents a true reduction in left ventricular function. Furthermore, although post-race HR increased significantly compared to pre-race HR, the absence of any significant correlation between HR and any Doppler-derived index of diastolic function supports the presence of a change in diastolic function.

Although our results agree with previously reported results, they should be interpreted with care. We report a weight loss of over 3%, which can be interpreted as a high degree of dehydration. We used weight changes and LVIDd as cardiac preload indicators; using weight loss as a preload indicator is open to question, however, as preload may be maintained by internal fluid shifts from the extravascular to intravascular space [29].

Moreover, although LVIDd may have been used as a measure of left ventricular preload in some studies [6,30,34], it is important to note that this parameter has limited use as a preload surrogate, given that the internal diameter of the left ventricle is inherent to the calculation of the LVEF.

Although our study has limitations because the parameters used to measure diastolic function are load-dependent, our findings agree with other studies that reported a reduction in diastolic function using a less load-dependent measure of diastolic function (tissue Doppler imaging and 2D strain analysis). Changes in diastolic function following exercise were unrelated to changes in preload-related indexes (LVIDd and haematocrit) and HR. Even in a controlled laboratory environment, where fluid ingestion was monitored throughout exercise and loading conditions were restored during the post-exercise assessment period, a decline in left ventricular diastolic function was observed. This suggests that the alterations in left ventricular relaxation are probably related to other underlying mechanisms [35].

Hence, as per Shave et al. [6], investigators may wish to use tissue Doppler imaging or 2D strain analysis to gain a better understanding of global left ventricular function after prolonged exercise, independently of left ventricular loading conditions.

The significant increase in highly specific cardiac injury biomarkers like cTnI and NT-proBNP observed in this study indicates that myocardial damage may have occurred as a result of prolonged strenuous exercise [1]. Damage to myocardial cells may cause the changes in cardiac function reported in several studies [21,22,36,37]. Nevertheless, it is not clear whether the changes in function or the release of biomarkers represent dysfunction or represent damage [37]. Moreover, according to most previous studies [6,28,37,38], the release of biomarkers and the changes in left ventricular function observed after prolonged strenuous exercise may be two concomitant, yet independent, phenomena.

## Conclusions

A long-duration cycling event led to a reduction in left ventricular systolic and diastolic function in cyclists and the unrelated appearance of elevated serum markers of cardiac myocardial cell damage. Nonetheless, the mechanisms behind these outcomes remain unknown. It is likely that factors other than myocardial damage contributed to the decrease in left ventricular function after intense exercise.

## Abbreviations

cTnI: Cardiac troponin I; IVSDd: Interventricular septum thickness during diastole; LVEF: Left ventricular ejection fraction; LVIDd: Left ventricular internal dimension during diastole; LVIDs: Left ventricular internal dimension during systole; LVPWd: Left ventricular posterior wall thickness during diastole; NT-proBNP: N-terminal proBNP

## Competing interests

The authors declare that they have no competing interests.

## Authors’ contributions

ESO conceived the study, participated in its design and coordination and helped draft the manuscript. MLR carried out tests, participated in designing the study and performed the statistical analysis. DCA carried out tests and assays and participated in designing the study. SIA helped draft the manuscript, revised it critically for intellectual content and gave final approval of the version to be published. PCE carried out the assays and participated in designing the study. CB and ALA participated in the sequence alignment and helped draft the manuscript. All authors read and approved the final manuscript.
